# High throughput single cell metagenomic sequencing with semi-permeable capsules: unraveling microbial diversity at the single-cell level in sewage and fecal microbiomes

**DOI:** 10.3389/fmicb.2024.1516656

**Published:** 2025-02-04

**Authors:** Meilee Ling, Judit Szarvas, Vaida Kurmauskaitė, Vaidotas Kiseliovas, Rapolas Žilionis, Baptiste Avot, Patrick Munk, Frank M. Aarestrup

**Affiliations:** ^1^Research Group for Genomic Epidemiology, National Food Institute, Technical University of Denmark, Kgs Lyngby, Denmark; ^2^Atrandi Biosciences, Vilnius, Lithuania; ^3^Institute of Biotechnology, Life Sciences Center, Vilnius University, Vilnius, Lithuania; ^4^Imperial College London, London, United Kingdom

**Keywords:** single cell sequencing, metagenomics, microbial diversity, sewage, antimicrobial resistance (AMR), pig fecal sample

## Abstract

Single-cell sequencing may serve as a powerful complementary technique to shotgun metagenomics to study microbiomes. This emerging technology allows the separation of complex microbial communities into individual bacterial cells, enabling high-throughput sequencing of genetic material from thousands of singular bacterial cells in parallel. Here, we validated the use of microfluidics and semi-permeable capsules (SPCs) technology (Atrandi) to isolate individual bacterial cells from sewage and pig fecal samples. Our method involves extracting and amplifying single bacterial DNA within individual SPCs, followed by combinatorial split-and-pool single-amplified genome (SAG) barcoding and short-read sequencing. We tested two different sequencing approaches with different numbers of SPCs from the same sample for each sequencing run. Using a deep sequencing approach, we detected 1,796 and 1,220 SAGs, of which 576 and 599 were used for further analysis from one sewage and one fecal sample, respectively. In shallow sequencing data, we aimed for 10-times more cells and detected 12,731 and 17,909 SAGs, of which we used 2,456 and 1,599 for further analysis for sewage and fecal samples, respectively. Additionally, we identified the top 10 antimicrobial resistance genes (ARGs) in both sewage and feces samples and linked them to their individual host bacterial species.

## Introduction

Advances in metagenomic sequencing techniques and bioinformatics approaches have significantly expanded our ability to explore the complex ecosystems of the microbial world, revealing the functional diversity and taxonomic composition of these communities (Jansson et al., 2012). Metagenomic approaches, while providing valuable information about the average characteristics of mixed microbial samples, have limitations resolving the true intricacies of these complex communities.

They might obscure the significant variability between individual bacterial cells when assembling genomes from mixed DNA populations obtained through bulk metagenomic sequencing (Liu et al., 2022; Quince et al., 2017). Microbial diversity can thus be underestimated through metagenomics, hindering the identification of individual species or strains within a community (Quince et al., 2017).

Single-cell sequencing addresses this limitation by sequencing DNA from individually barcoded cells. By utilizing high-throughput single cell sequencing, the technology provides insights into the intricate composition of the bacterial communities at the individual cell level (Hosokawa et al., 2022; Sharma and Thaiss, 2020). This enables a detailed exploration of the genetic diversity and functional roles of microorganisms at the single-cell level, revealing strain heterogeneity and identifying novel taxa within complex matrices. It also offers a deeper understanding of the functional contributions of individual cells (Blainey, 2013). Thus, single-cell DNA sequencing has the potential to reveal the trends of dispersal of antimicrobial resistance genes (ARGs) in the context of real-life environmental and host microbiome monitoring.

Droplet microfluidics, which enables the capture of hundreds of individual cells per second, is an attractive technology to satisfy the throughput requirement, and explore the depths of microbial diversity in complex ecosystems (Lan et al., 2024; Tolonen and Xavier, 2017; Hosokawa et al., 2017; Zheng et al., 2022; Hosokawa et al., 2022). However, the use of liquid droplets as single cell encapsulation for single-microbial cell DNA sequencing applications comes with challenges. Once a droplet forms, its contents can only be diluted, not entirely exchanged, which limits flexibility in subsequent processing steps like waste exchange as an example. In addition, lysis, amplification, and barcoding reagents within each step must be compatible, which limits their choice. Furthermore, reagent addition to droplets is practically challenging and requires training in advanced droplet manipulations such as re-injection, pico-injection or droplet merging.

Here, we demonstrate the use of the semi-permeable capsules (SPCs) technology (Leonaviciene et al., 2020) for single-microbial cell DNA sequencing. SPCs enable multistep workflows on thousands of individual genomes in parallel without the constraint of reaction compatibility, because their content below a given molecular size cut-off can be easily and fully replaced. In addition, this innovative approach overcomes laborious techniques of droplet manipulation and increases the throughput of single-cell genome analysis. In our study, we applied this new single cell microbiome sequencing method to investigate the microbial diversity and distribution of ARGs in individual microbial cells from sewage and pig fecal samples and compared it with shotgun metagenomic data. Furthermore, we explored the sequencing information that can be obtained through deep sequencing (a lower number of bacterial cells with a higher number of reads) and shallow sequencing (a higher number of cells with a lower number of reads) with this microfluidic single-cell isolation and SPCs sequencing method.

## Methods

### Sample collection and preparation

A sewage sample from Bangladesh in 2018 (Bgd01) (Munk et al., 2022) and a pig fecal sample collected in Denmark in 2019 on 14/05–2019 (# 1166, Week 1, pig E, W1E for short) were used. Samples were stored in −80°C freezer prior to DNA extraction and single-cell encapsulation in SPCs. The third sample sequenced was the sewage sample (Bgd01) that was spiked with 100 μL of ZymoBIOMICS^®^ Gut Microbiome Standard (No. D6331) containing 14 bacterial and archaeal species to 0.1 g of the sewage sample before the SPCs prep procedure.

### Preparation of detached bacterial cells from the environmental sample for single cell encapsulation

A modified version of Morono’s cell detachment protocol was used for this experiment (Morono et al., 2013). The modifications of the protocol were as follows. 0.1 g of sewage or feces sample was suspended in 150 μL of a 2.5% NaCl solution. Subsequently, 50 μL of a detergent mix [100 mM EDTA, 100 mM sodium pyrophosphate, 1% (v/v) Tween 80] and 50 μL of methanol were added to the suspension, and the sample was vigorously shaken for 60 min at 500 r.p.m. using a shaker. Following the shaking step, the sediment slurry was sonicated three times for 1 min each in a water bath. Next, 1 mL of a 2.5% NaCl solution was added to the samples, and the mixture was filtered through an 8uM-sized filter syringe. The collected supernatant was then centrifuged at 15,000 × g for 10 min, after which the supernatant was removed. The cell pellets were suspended in 1x PBS, and washed twice at 8,000 × g for 5 min.

### Generation of SPCs

Before single-cell encapsulation, the total number of cells in the sewage and fecal samples was measured using impedance flow cytometry (Bactobox, SBT instrument). The measured total number of bacterial cells from each sample was used to achieve lambda of 0.1 by calculating the prepared sewage and fecal samples concentration for droplets loading.

SPCs were produced on the ONYX platform (Cat no CHN-ONYX2, Atrandi Biosciences) using the SPCs innovator kit (Cat no CKN-G11, Atrandi Biosciences). Core and shell solutions were prepared according to the manual, targeting 0.1 cells/SPC (lambda value) to prevent the encapsulation of multiple cells in a single capsule. After 1 h of encapsulation, the emulsion was collected in a 1.5-ml Eppendorf tube and the shells were cross linked using the Light Exposure Device (Cat no MHT-LAS1, Atrandi Biosciences). Next, the oil phase was removed by aspirating the bottom phase with a 1-ml pipette and SPCs were recovered by breaking the emulsions with the Emulsion Breaker solution provided in the kit and suspended in aqueous buffer supplemented with 0.1% Triton X-100. In this study, roughly 100,000 cells were encapsulated for each sewage and fecal sample, and around 50,000 cells per sample was used for the combinatorial barcoding step, based on the calculation of the occupancy value from the fluorescent images after DNA amplification.

### Cell lysis protocol

Individual cells in SPCs were lysed in two steps. In the first step, SPCs were incubated in 1 mL mixed lysis solutions (50 U/μL Lysozyme Solution (Epicentre), 2 U/mL Zymolyase (Zymo research), 22 U/mL lysostaphin (MERCK), and 250 U/mL mutanolysin (MERCK) in PBS) at 37°C overnight. Then, the SPCs were washed with 1 mL of 1x PBS three times to replace the reagent. The second lysis treatment involved the 1 mL of 1 mg/mL Proteinase K (Promega) in PBS at 40°C overnight, after which the SPCs were washed three times with PBS. 500 μL of SPCs were resuspended in the alkaline lysis solution (final concentration 0.4 M KOH, 10 mM EDTA, 100 mM DTT) and spun down after 15 min incubation at room temperature. Following the previous alkaline lysis step, the SPCs were washed three times with 1 mL of neutralization buffer (1 M Tris–HCl (pH 7.5)) and three times with 1 mL 10 mM Tris–HCl (pH 7.5) with 0.1% Triton X-100.

### DNA amplification and combinatorial barcoding

Whole genome amplification and combinatorial barcoding were performed with a custom Single- Microbe DNA Barcoding Kit (Cat no CKP-BARK1, Atrandi Biosciences). Briefly, SPCs were incubated with the Whole Genome Amplification (WGA) mix at 45°C for 1 h and the reaction was inactivated at 65°C for 10 min. Following WGA, SPCs were washed three times with 500 μL of 10 mM Tris–HCl (pH 7.5) with 0.1% Triton X-100. Thereafter, SPCs were stained with 1× volume SYBR Green (Thermo Fisher Scientific) in 1x PBS to confirm DNA amplification by the presence of green fluorescence in the capsules by fluorescent microscopy. Microscope images for SPCs after WGA were recorded to estimate the single-amplified genome (SAG) occupancy in the SPCs, i.e., the fraction of fluorescent capsules. After this quality control step, SPCs underwent DNA debranching and end preparation according to the manual. A four-step combinatorial split-and-pool barcoding was performed to label DNA fragments with SPCs-specific sequences. We used 16 × 96 × 96 × 96 barcode combinations producing a diversity of ~14 M unique variants. The 16-variant barcode (referred to as “barcode D”) is added first, as it is used to identify the sample itself in a multi-sample analysis scenario. In our study, barcode D variants 1–8 were used to label the fecal sample and barcode D variants 9–16 the sewage sample.

### Library preparation for Illumina sequencing

The SAG sequencing libraries were prepared from sub-aliquots of SPCs containing barcoded genomes from the pool of sewage and fecal samples. SPCs were dissolved using the Release Reagent (Atrandi Biosciences) and barcoded DNA was purified with 0.8X Ampure XP (Beckman Coulter) paramagnetic beads. Next, the NGS library was prepared using the NEB Next® Ultra™ II FS DNA Library Prep Kit for Illumina (Cat. No E7805S, NEB) and custom PCR indexing primers (Supplementary chart 1, IDT) according to the instructions provided in Single-Microbe DNA Barcoding Kit. The obtained libraries were quantified with Agilent High Sensitivity DNA Kit (Cat. No. 5067–4,626, Agilent). Two different libraries (deep sequencing and shallow sequencing) from the same encapsulation sample were created. For the deep sequencing approach, 400 SPCs from fecal sample (W1E) and 400 SPCs from the sewage sample (Bgd01) were combined for the last DNA purification step for a total of 800 SPCs targeting 60 GBp. For the shallow sequencing method, 4,000 SPCs from fecal sample (W1E) and 4,000 SPCs from the sewage sample (Bgd01) also targeting 60 GBp reads. Libraries were loaded at 1.8 pM concentration for sequencing on the Illumina NextSeq550 platform with High Output Kit v2.5 (300 cycles). Sequencing performed with the following read lengths: R1–134 bp, R2–178 bp, i7 – 6 bp and the raw sequence data (.bcl files) were used as input for further processing.

### Basecalling and de-multiplexing the sequencing data from the SAG libraries

The bcl files from each flow cell were basecalled using Illumina bcl2fastq2 Conversion Software v2.20^1^ with the default settings. Each pair of FASTQ files were thereafter de-multiplexed based on their barcoding plate well-specific barcode, sequenced on the reverse read, using Ultraplex v1.2.5 (Wilkins et al., 2021). The supplied barcodes were formatted as a list of the 16 possible 8-mer oligos and padded with Ns on the 5′ end to the total length of 44 bases. In each barcode, a maximum of one mismatch was allowed.

To estimate the observed number of cells (Supplementary chart 1), the barcode-bearing parts (B, C, D) of the resulting FASTQ files, after demultiplexing by barcode A (Supplementary chart 1, step 1), were analyzed. Briefly, unique A-B-C-D combinations were counted, and the results were plotted as a weighted histogram in Python (plt.hist(np.log10(reads_counts_per_bc_combination), weights = reads_counts_per_bc_combination)). Note that in this analysis, barcode A is matched against the expected whitelist, while barcodes B, C, and D are analyzed in a whitelist-agnostic manner.

The raw FASTQ files were trimmed in two steps using fastp v0.23.2 (Chen et al., 2018). In the first pass, the TruSeq adapters were removed, and the reads were trimmed to have a minimum average quality score of 30 (99.9%) and a minimum length of 100. Afterwards, the reverse reads were split into the genomic fragment and the barcode, with the three 8-mer oligos being excised and concatenated together. Thereafter, the forward read and the genomic fragment containing reverse read were again processed with fastp to remove any barcode from the 3′ end of the forward reads, using the default overlap analysis method and the quality and length filtering disabled.

The trimmed paired-end FASTQ files were further de-multiplexed into individual SAGs via their SPCs-specific barcode-triplets using *high_dimensional_demultiplexing.py* (https://bitbucket.org/genomicepidemiology/hitesc, commit: 1d71f7e). The input parameters for sub-sampling (*n* = 0.005r) and minimum read counts (c = 0.0005n and 0.0003n) were derived from the fastp-reported total trimmed read counts (r). For the annotation steps, the sewage and feces SAGs were filtered based on the number of reads for each SAG. We excluded SAGs with more than 1 million reads for both the shallow and deep sequenced SPCs, and fewer than 50,000 for the sewage and fewer than 70,000 reads for the feces SAGs for deep sequencing. The spike-in sample derived SAGs with less than 60,000 reads were filtered out.

### Mapping the spike-in sample to its reference genomes

Trimmed reads were mapped to the reference genomes of the Gut Microbiome Standard (D6331) using KMA v1.4.12a (Clausen et al., 2018) with the parameters `-ID 0.50 -apm f -mrs 0.84 -ml 100`.

### Pre-processing the sequencing data from the metagenomic libraries

The sewage and the pig fecal sample was also shotgun metagenomic sequenced, as described in Munk et al. (2022). The paired-end FASTQ files for the metagenomes were adapter- and quality trimmed with BBduk2 script from BBMap v36.49 (Bushnell, n.d.). The quality trimming was set to remove bases on the 3′ end below phred score of 20, using a sliding window.

### Taxonomic profiling and antimicrobial-resistance gene prediction

The individual trimmed FASTQ files for the SAGs and the metagenomes were profiled using MetaPhlAn v4.0.6 (Blanco-Míguez et al., 2023) with the default settings and database vOct22. Taxonomic annotation was accepted, if the mapped reads’ relative abundance was larger than 40%. For the antimicrobial-resistance gene prediction, we used KMA v1.4.12a (Clausen et al., 2018) with the parameters ` -ID 45.0 -bc 0.5 -mrs 0.90 -ml 75 -cge` aligning reads to the PanRes database v1.0.0 (Martiny et al., 2024). The hits were summed to both homologous reference gene clusters with 90% identity, and antimicrobial classes as provided in PanRes metadata.

## Results

### Semi-permeable capsules enabled the sequencing of tens of thousands of individual genomes

**Figure 1:**
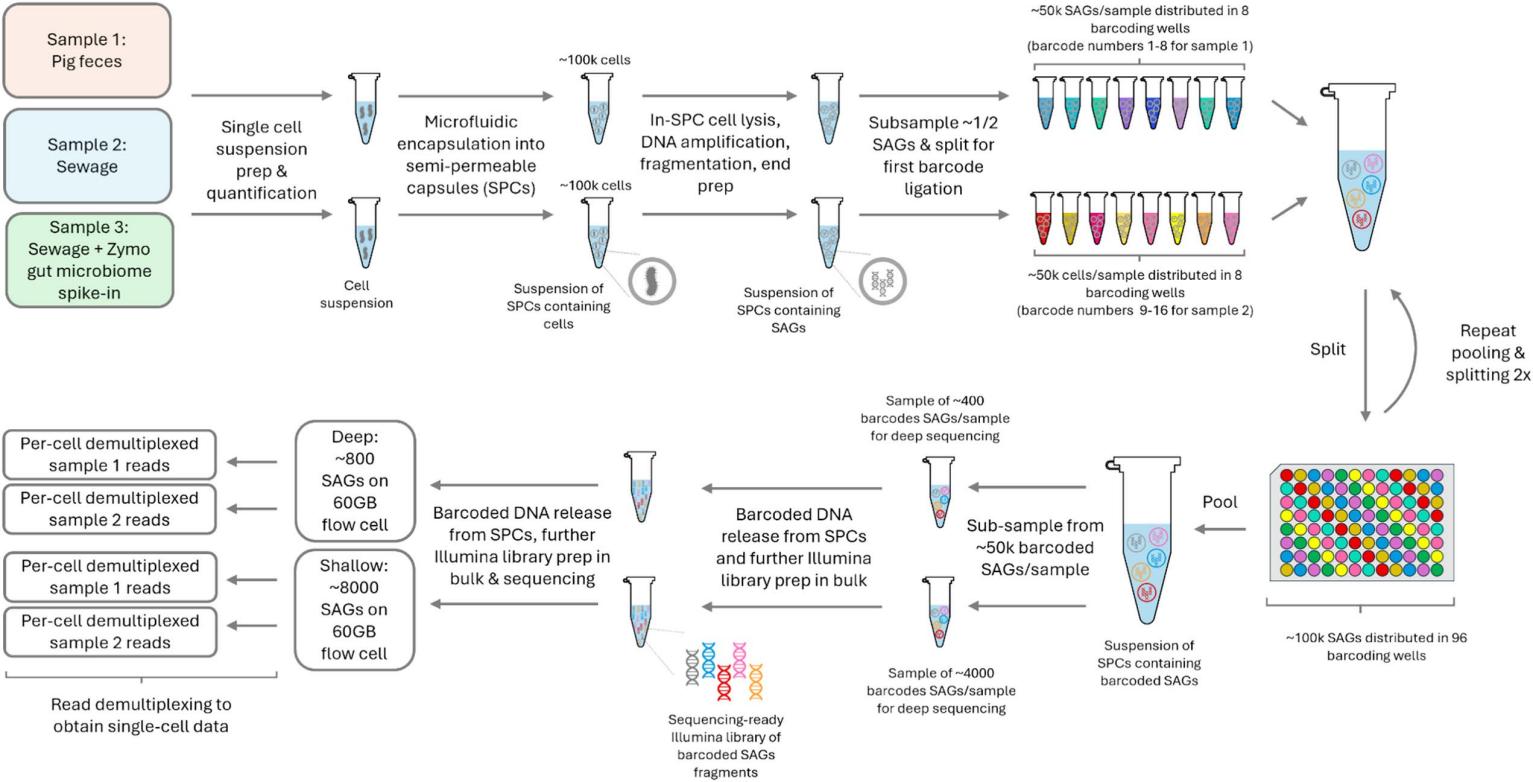
Experiment outline from sample to demultiplexed per-cell sequencing data. The figure highlights the workflow for both the pig feces and sewage samples. SPC – semi-permeable capsules; SAG – single amplified genome.

Following the cell detachment protocol from sewage and fecal samples, single bacterial cells were isolated in semi permeable capsules (SPCs) (65-70 μm). In our procedure, roughly 100,000 cells were encapsulated for each sewage and fecal sample, as estimated from a digital enumeration of fluorescent SPCs post-MDA and DNA staining. Around 50,000 single-amplified genomes (SAGs) per sample underwent a ligation-based combinatorial split-and-pool barcoding, preceded by a fragmentation and DNA end-prep step. Post-barcoding, the content of SPCs was released and pooled, followed by further steps for Illumina pair-end sequencing library preparation (Figure 1).

### Spike-in sewage sample

In order to demonstrate the capability of the method for capturing genomic DNA from cells in a complex biological matrix, we spiked cells from a gut microbiome standard (Zymo) into a sewage sample (bgd01) before processing it. After sequencing and demultiplexing to individual SAGs, 60.4% of the trimmed reads were assigned to SAGs with more than 60,000 reads each. Our anticipated number of SAGs was 4,000 for this experiment, of which we captured 2,330.

We mapped the trimmed reads to the reference genomes of the cells that were spiked in to verify the efficiency of the DNA extraction and amplification methods. We found that the procedure could amplify both Gram-positive and Gram-negative genomes, however Gram-negative species in the mock community had consistently lower depth of coverage than the Gram-positive cells of the same proportion of genomic DNA (Supplementary Table 1). Note that metagenomic applications, for which the standard was designed and that rely on whole DNA extraction, are unaffected by Gram-negative population lysis.

### Single amplified genomes (SAGs)—shallow sequencing and deep sequencing

**Figure 2:**
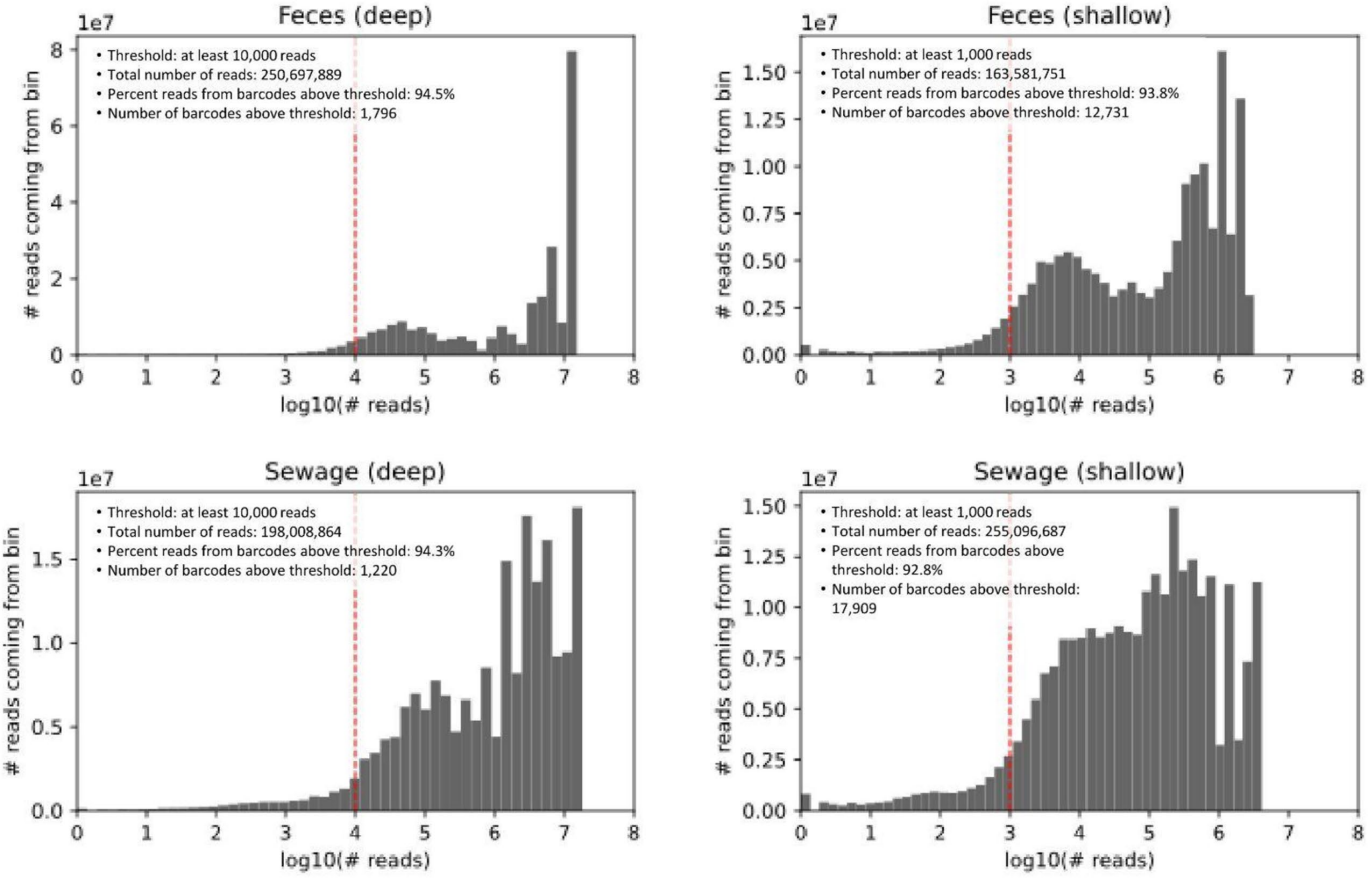
Weighted histograms of reads-per-barcode. Barcodes are binned by the number of reads, and the y-axis shows the number of reads coming from each bin. The gray area in the figure is proportional to the number of reads.

Library sizes (targeting 400 SAGs for deep sequencing and 4,000 SAGs for shallow) were estimated by digitally enumerating fluorescent SPCs post-MDA (see Methods). The observed number of cells per library was determined from weighted barcode abundance histograms (Figure 2). In these histograms, barcodes are binned by read number, with the y-axis representing the number of reads per bin. This representation offers two key advantages. Firstly, it allows for visual identification of a natural threshold to distinguish target SAGs from noise, such as any trace barcode cross-contamination between wells Secondly, since the histogram area is directly proportional to the number of reads, it emphasizes the economic impact of thresholding.

The four libraries received between 164 M and 255 M reads. By design, deep sequencing libraries were expected to contain about 10-fold fewer cells. When comparing deep vs. shallow libraries, the distributions were expected to have similar shapes but be shifted roughly 10x on the x-axis. Both the consistent distribution shape and the approximate 10x shift on the x-axis were observed (Figure 2). A bimodal distribution was observed for both sample types, with a more pronounced separation in the fecal samples. The lower read count subset may represent smaller genomes (such as phages) or partial genomes, either due to fragmentation during sample preparation and storage or incomplete lysis.

As shown in Figure 2, we visually selected thresholds of 10,000 reads for deep sequencing libraries and a 10-fold lower threshold for shallow libraries. In deep sequencing libraries, this threshold identified 1,796 and 1,220 barcodes (SAGs) for feces and sewage, respectively. In shallow sequencing, the SAG counts were 12,731 and 17,909, respectively—3 to 4 times more than anticipated from the digital quantification of fluorescent SPCs. This discrepancy highlights the challenge of estimating SAG numbers in environmental samples, where genome sizes and the corresponding amount of amplified DNA can vary across several orders of magnitude. A possible explanation for underestimating SAG counts using digital quantification is the difficulty in distinguishing between empty SPCs and those containing low amounts of DNA.

For taxonomic annotation, antibiotic resistance gene analysis, and comparison to metagenomic data, we proceeded with a minimum of 70,000 reads per SAG for feces and 50,000 reads per SAG for sewage in the deep sequencing libraries (Supplementary Figure 1). The threshold for shallow sequencing libraries was automatically scaled for each barcode A variant during demultiplexing (parameter c=0.0005n ; see Methods) and ranged from ~9,000 to 14,000 reads for feces and from 5,000 to 24,000 reads for sewage. This resulted in 599 and 576 cells (deep sequencing), and 1,599 and 2,456 cells (shallow sequencing) being used for further analysis of sewage and fecal samples, respectively.

### Top taxonomic labels of bacteria agree between shallow and deep sequencing

The five most abundant species in the pig fecal sample using the shallow sequencing method were: *Lactobacillus amylovorus* (412 SAGs), *Mogibacterium kristiansenii* (96 SAGs), *Streptococcus alactolyticus* (89 SAGs), *Limosilactobacillus reuteri* (76 SAGs) and *Streptococcus suis* (38 SAGs) (Supplementary data). The top five dominant species for deep sequencing were similar, with the exception that *Streptococcus suis* was replaced by a *Clostridial* bacterium (33 SAGs). However, *Streptococcus suis* still remained one of the top ten dominant species in deep sequencing.

For the sewage sample, the five most abundant species using the shallow sequencing method were: *Streptococcus suis* (404 SAGs), *Planococcus plakortidis* (113 SAGs), *Planomicroboium koreense* (61 SAGs), *Streptococcus equinus* (59 SAGs), *Planococcus rifietoensis* (51 SAGs) (Supplementary data). The five most abundant species identified through shallow sequencing closely resemble those found using the deep sequencing method. For example, *Streptococcus suis* (189 SAGs) remained the most dominant species while *Planococcus plakortidis* (12 SAGs) and *Streptococcus equinus* (10 SAGs) were the next two dominant species found in the deep sequencing approach. With the exception that *Planomicrobium koreense* is not the dominant species, the dominant species is replaced by *Bifidobacterium longum (9 SAGs)* and *Bifidobacterium adolescentis (8 SAGs)* which are tw*o Bifidobacterium* sp. that commonly found in the human gut microbiota and *Ligilactobacillus ruminis* (8 SAGs).

**Figure 3:**
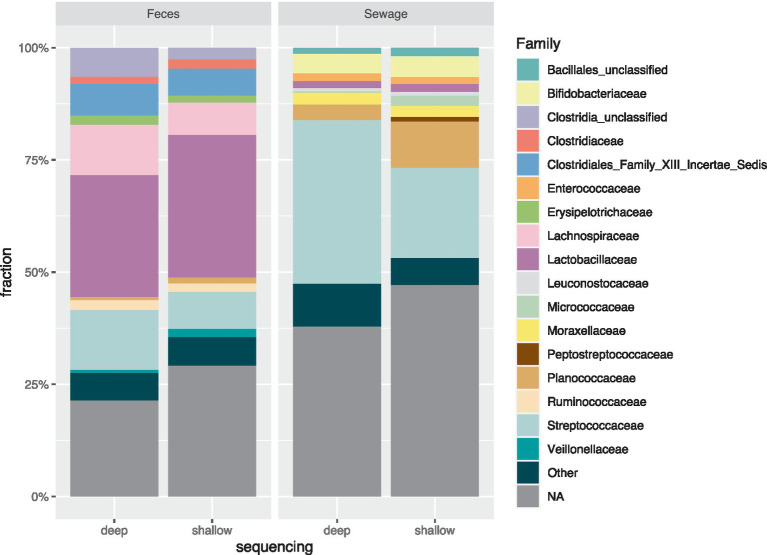
Taxonomic distribution at family level for both shallow sequencing and deep sequencing from sewage and pig feces, showing the top 10 families in each sample. Top 10 families from each sample are plotted across all 4 bars. Dark green color for “Other” category contains the families that were not in the top ten in the samples. The NA category encompasses the droplets where taxonomy on the family level could not be assigned.

In general, deep sequencing of the fecal sample revealed a lower count of bacterial species but not number of families. Especially *Streptococcaceae* and *Lachnospiraceae* were common (Figure 3). For both samples, a higher fraction of SPCs assigned to *Streptococcaceae* were seen in deep sequencing as compared to shallow sequencing. Shallow sequencing of the sewage sample showed higher proportions of cells in the *Enterococcaceae* family.

### Read based ARG prediction for deep and shallow sequencing

Acquired antimicrobial resistance genes were predicted in the SAGs using the PanRes database. The ARG with the highest hit count in both samples was *erm*B (U86375), which confers resistance to macrolides and lincosamides.

**Figure 4:**
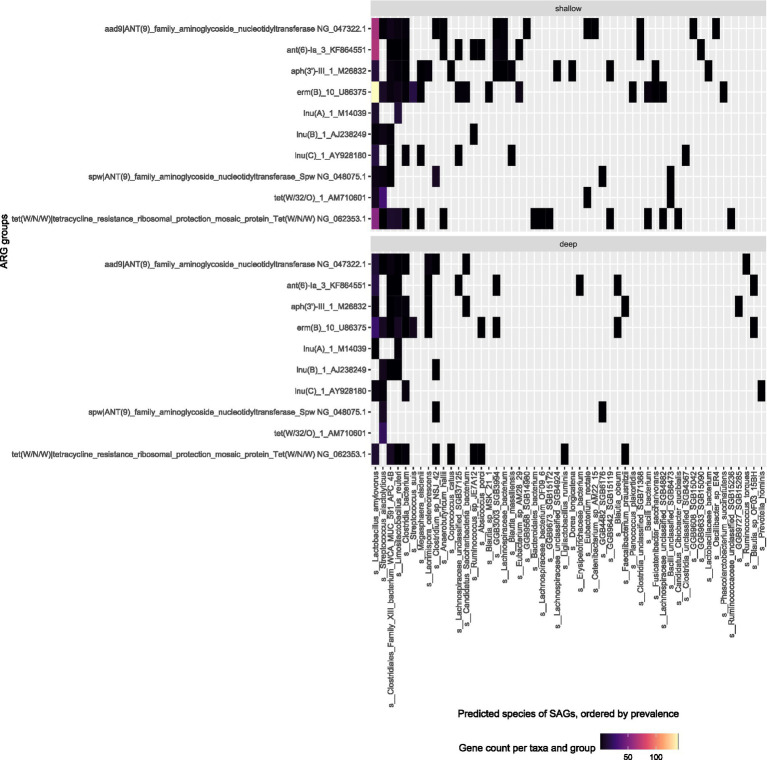
Top ARG groups in pig fecal sample (W1E) and the predicted species of the SAG they were found in. Top: Shallow sequencing; Bottom: Deep sequencing.

Our results showed that the shallow sequencing approach detects more ARGs than the deep sequencing in the more prevalent bacterial species. In the pig feces sample (Figure 4), *L. amylovorus* (412 SAGs in shallow sequencing) was the dominant bacterial taxon, and it was associated with the most frequently detected ARGs: it harbored all but three of the top 10 ARG groups found via deep sequencing, and all the top ARG groups are attributed to it by the shallow sequencing method. Similarly, the deep and shallow sequencing approaches both identified six major ARGs in *S. alactolyticus.*

**Figure 5:**
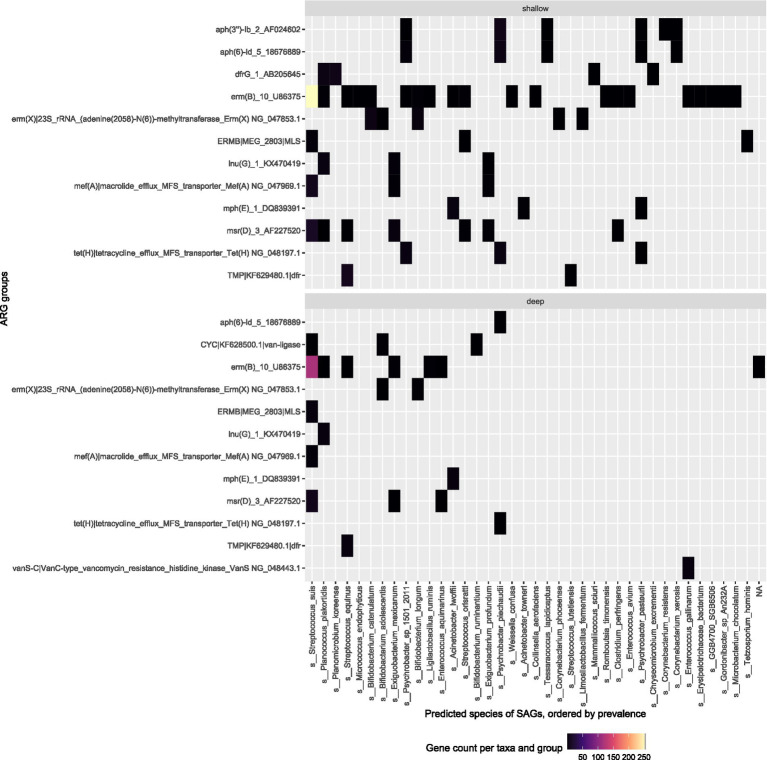
Top ARG groups in the sewage (Bgd01) sample and the predicted species of the SAGs they were found in. Top: Shallow sequencing; Bottom: Deep sequencing.

In the sewage sample, a similar trend is observed; the second and third most prevalent species *Planococcus plakortidis* and *Streptococcus equinus* revealed more ARGs in shallow sequencing as compared to deep sequencing (Figure 5). The deep sequencing approach only revealed more ARGs compared to shallow sequencing in those bacterial species that are less prevalent within the samples such as *Enterococcus aquimarinus, Enterococcus gallinarum,* or *Bifidobacterium adolescentis.*

### Comparisons between metagenomic data, shallow and deep SAGs: taxonomic profiles and ARGs

We performed a comparison of taxonomic assignments and ARGs based on (1) shallow SAGs, (2) deep SAGs and (3) metagenomics, separately for feces and sewage. We only sequenced SAGs from a few thousands of cells, whereas the genomic data obtained through shotgun metagenomics data results from sequencing from cells, so the two approaches have distinct advantages and disadvantages.

In pig feces, one extra family was found by deep sequencing compared to shallow sequencing: Propionibacteriaceae. We detected 13 bacterial families exclusively in shallow sequencing (unclassified Bacillales, Carnobacteriaceae, Corynebacteriaceae Dietziaceae, Enterococcaceae, FGB3048, FGB39849, FGB8240 unclassified Lactobacillales, Methanosarcinaceae, Micrococcaceae, Ornithinimicrobiaceae, Peptostreptococcaceae), owing to the fact, that the shallow sequencing method sequences more bacterial cells from the same sample and provides insight into larger bacteria diversity.

In the sewage sample, bacteria in four bacterial families were unique to the deep sequencing method: Brucellaceae, Bradyrhizobiaceae, Legionellaceae and Peptococcaceae. On the other hand, 11 bacterial families were uniquely detected by shallow sequencing (Bacillaceae, unclassified Bacilli, Chromatiaceae, Dermacoccaceae, Dermatophilaceae, FGB7832, Hungateiclostridiaceae, Ornithinimicrobiaceae, Rhizobiaceae, Sphingobacteriaceae, Vibrionaceae) (Supplementary data).

**Figure 6:**
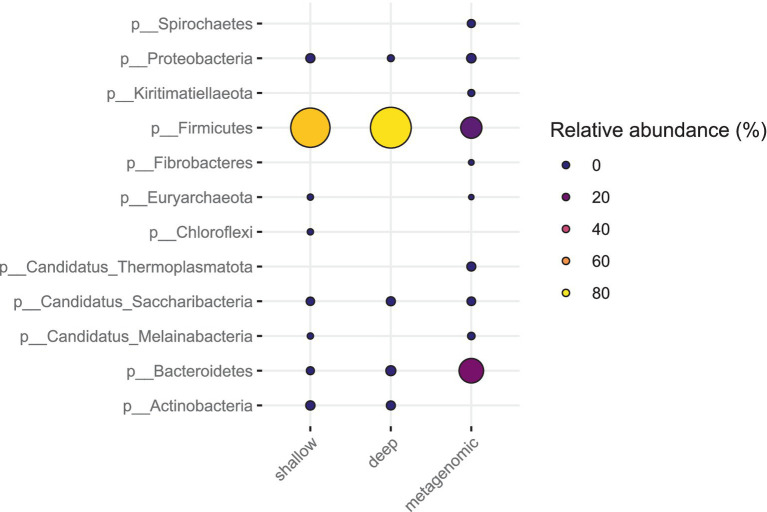
Bacterial phyla of the gut microbiota in pig feces (W1E) from deep sequencing, shallow sequencing and metagenomic sequencing. The coloring and size of the circles correspond to the relative abundance of each phylum.

**Figure 7:**
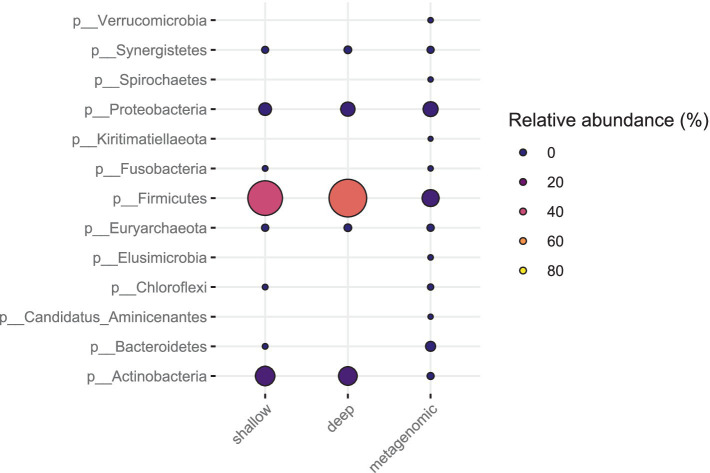
Bacterial phyla from sewage sample (Bgd01) from deep sequencing, metagenomic and shallow sequencing. The coloring and size of the circles correspond to the relative abundance of each phylum.

The relative abundance estimates from metagenomic sequencing and the SAG counts from SC sequencing did not correlate well for all phyla and families (Figures 6, 7). For the fecal sample, *Prevotella* was the most abundant species in metagenomic data, while the most abundant species in SC data was *Lactobacillus amylovirus*. Interestingly, only two SAGs of *Prevotella* were found using the SC method. Our mock community analysis likewise revealed that the SC method captured more Gram-positive bacterial DNA than Gram-negative genomic DNA. This is consistent with the higher relative abundance of the phylum *Bacteroidetes* (Gram-negative) observed in metagenomic data compared to our SC protocol in feces. The single cell sequencing method revealed two phyla in the pig fecal sample that metagenomic sequencing did not recover: Actinobacteria and Chloroflexi (Figure 6). For the sewage sample, the phyla that were identified by single cell sequencing are also found in metagenomic sequencing.

**Figure 8:**
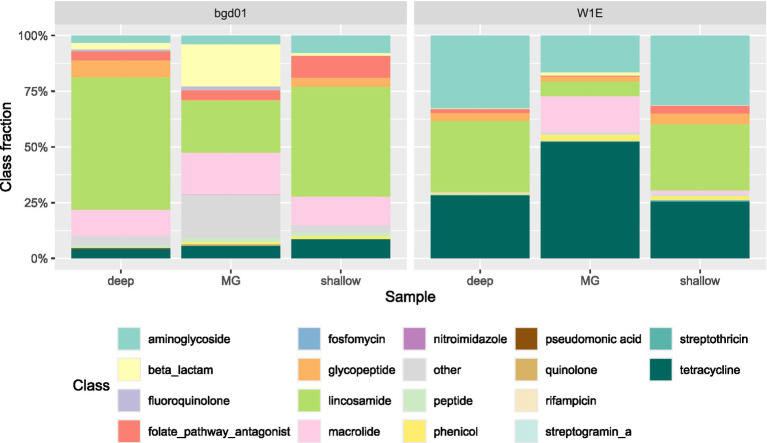
ARG phenotypic distribution for the SC deep sequencing, SC shallow sequencing and MG data for pig feces (W1E) and sewage (bgd01). Fractions are calculated on the total number of ARGs or reads found in each sequencing run.

The dominant class of resistance in the sewage sample, by both shallow and deep sequencing, is lincosamide (Figure 8), while tetracycline was the most frequently found resistance class based on metagenomic analysis. Similarly, in the pig fecal sample, lincosamide was the dominant class that was found in SAGs and tetracycline was the dominant resistance class found in the metagenome data.

## Discussion

Shotgun metagenomic sequencing of environmental samples has made it possible to profile entire microbial communities. Combining recent advancements in microfluidics and SPCs technology, direct genomic analysis of individual microbial cells within complex environmental samples, called single cell metagenomic sequencing, is now enabling the focused analysis of single members of complex communities, without the bias of pre-selection (Lan et al., 2024). Our study demonstrated a laboratory platform that can access high-throughput single cell metagenomic sequencing techniques including single cell isolation from sewage and pig feces microbiomes, genome amplification in SPCs techniques and split-pool ligation-based barcoding of tens of thousands of individual SAGs in parallel.

Single bacterial cells were isolated in 65-70 μm SPCs that have a liquid core surrounded by a thin semi-permeable shell, which retains DNA fragments >500 bp but allows enzymes, oligos and smaller molecules to diffuse through (Leonaviciene et al., 2020). Thus, encapsulated cells can lyse in a lysis mix and process through series of washing steps to purify and amplify the cells’ genetic material (Leonaviciene et al., 2020). To mitigate DNA losses during further library preparation steps, the purified DNA was amplified by multiple-displacement amplification (MDA) within SPCs. To ensure single cell encapsulation of bacteria within environmental samples, the total bacterial count within a sample is important for the precalculated lambda values prior to encapsulation. Due to the variability of the initial measurement of bacteria number within an environmental sample that might be influenced by bacterial cell clusters, the precalculated SAGs used for sequencing might not represent the final number of SAGs that are sequenced (Figure 1).

We used MetaPhlAn4 with individual SAGs as input to predict the taxonomical profile of the sequenced microbial communities. However, reference databases are not expected to contain all strains found in all types of environments and host microbiomes. Microbial dark matter, made up of yet un-discovered or un-described taxa, could make up a significant portion of a complex environmental community. Thus, identifying all our SAGs from complex microbial communities down to the species level through comparison with known reference genomes is not entirely feasible. More complete reference databases, or reference-free methods could potentially identify more unknown SAGs. Additionally, the limited coverage of the genomes can hinder taxonomic assignment, especially on the lower taxonomic levels. Despite these challenges, our single cell metagenomic sequencing method revealed a diverse range of bacterial species from various phyla both with deep and shallow sequencing.

The single cell sequencing method we introduce here demonstrated potential bias against Gram-negative bacteria (Supplementary Figure 2). The observed discrepancy could be attributed to the freeze–thaw process during sample storage or preparation protocol. Specifically, sample preparation steps like microbial cell detachment, which includes sonication and centrifugation steps may lyse Gram-negative cells before the cell encapsulation step. Therefore, the microbial community composition might be altered after post-extraction procedure. In contrast to the standard protocol for metagenomic library preparation, single cell sequencing also excludes the free-floating DNA that has been prematurely released from cells before cell encapsulation step. Therefore, samples that are used for single-cell DNA sequencing should be prepared or extracted using a procedure that has minimal impact on cell lysis before the cell encapsulation step. The results from the spiked sample are also consistent with previous attempts to use the Zymo reference standard for single-cell method evaluation, which report Gram-negative underrepresentation due to lysis caused by the DNA/RNA Shield reagent (Li et al., 2023; Lan et al., 2024). Future studies employing single-cell metagenomic protocols should modify the sample preparation steps to use alternative methods that avoid lysing bacterial cells before encapsulation. Additionally, it is crucial to ensure that the method can effectively detach bacterial cells from clustering, aggregating, or adhering to environmental particles.

In our single cell workflow, a single run of experiment can generate roughly 100,000 SPCs that each contain a single cell. Due to the limited sequencing capacity of flow cells, different number of SAGs sequenced in one flow cell would change the read number obtained per SAG (Figure 1). Therefore, different SC sequencing approaches can be used to serve various research objectives. The Deep sequencing method samples fewer bacteria, which reduces the bacterial diversity obtained but increases the number of reads per cell. More reads per SAG might provide a broader sampling of genetic material in one cell including antibiotic resistance genes (ARGs) and potentially improve taxonomy assignment accuracy for rare bacterial strains. In contrast, shallow sequencing yields higher bacterial diversity information within the sample by capturing more information across a higher number of SAGs, potentially making it useful to track specific ARGs across multiple bacterial species. It could also still provide detailed genetic insights into dominant bacterial species as the lower number of reads per cell can be compensated by the increased number of SAGs. However, the shallow sequencing approach may not effectively capture rare species. The two different sequencing approaches using the single cell barcoding workflow presented here should be adjusted based on specific research objectives.

The SC sequencing method has the potential to quantify abundance of specific bacterial taxa within a sample, relative to the number of representative encapsulated cells which reveal the presence of dominant species in environmental or fecal samples. The presence of dominant species like *Lactobacillus amylovorus*, *Streptococcus alactolyticus*, *Limosilactobacillus reuteri* in our pig fecal sample is in concordance with the results showing the top 5 most prevalent species found in a large-scale deep metagenomic sequencing study conducted on bacterial isolates from pig intestines (Wylensek et al., 2020). *L. amylovorus* is the most abundant species found at both the deep and shallow sequencing methods in pig fecal sample (145 SPCs, 412 SPCs). *L. amylovorus,* the most abundant species found in our pig fecal sample, is commonly found in pig feces and is known for its probiotic effect in piglets (Shen et al., 2022; Wylensek et al., 2020). Moreover, several species normally found within pig specific gut microbiota were also found to be abundant, such as species of *Lactobacillus*, *Streptococcus*, *Clostridium*, *and Enterococcus* (Wylensek et al., 2020). One of the newly cultured taxa that represents a dominant species from pig intestine described by Wylensek et al. (2020) is *Mogibacterium kristiansenii* which is the second most abundant species found in our single cell study. This result showed that single cell sequencing has the potential to reveal certain new bacterial taxa, complementing efforts to cultivate new bacterial taxa from specific environments or hosts.

Interestingly, in pig feces, SC sequence data from both deep and shallow sequencing revealed the presence of Actinobacteria, which was not found in the metagenomic sequencing of our sample (Figure 7). A previous study has shown that the core gut microbiota of piglets mainly consists of the bacterial phyla Firmicutes, Proteobacteria, Bacteroidetes, and two minority phyla: Actinobacteria and Fusobacteria (Cremonesi et al., 2022). Another study also showed that Actinobacteria is one of the three phyla that dominate piglet feces (Lührmann et al., 2021). This demonstrates the potential of SC sequencing to uncover certain bacterial phyla that cannot be discovered by certain metagenomic analysis unless one would make a thoroughly detailed bioinformatics analysis on certain metagenome sequence data.

The taxonomy assignment of both sequencing approaches reveals the dominant bacterial taxa within the samples while providing extra information that can directly link ARGs to each taxon and the distribution of the ARGs across multiple bacterial classes. Compared to metagenomic sequencing, the direct linking of specific genes from single-cell sequencing offers a significant advantage. It allows for tracking the spread of ARGs within the same environment and between different bacterial species. Both shallow sequencing and deep sequencing methods can identify ARGs within bacterial genomes in sewage and fecal samples (Figures 5, 6). In our study, we were able to identify the ARGs in *Streptococcus suis,* a bacterial pathogen associated with swine and human disease (Rayanakorn et al., 2018; Hughes et al., 2009), that dominates the Bangladesh open sewage systems. This demonstrates that single-cell sequencing can be used to determine antibiotic susceptibility profiles of specific bacterial taxa within the sample, which is crucial for mapping the epidemiological spread of ARGs of certain human pathogens in the environment that is close to human contact.

Twenty five bacterial species harbor the top ARGs found within the deep sequencing of the pig fecal sample and 48 bacterial species are represented in the top ARGs found in the shallow sequencing approach. Shallow sequencing will provide distribution of dominant ARGs across different bacterial taxon and species within the environment. The deep sequencing method is a better approach if the objective of study is to investigate ARGs within less dominant bacterial species while shallow sequencing approach is better for investigating the ARGs within the most dominant bacterial species. This was shown in the feces sample analysis where *L. amylovorus* associated with most frequent ARGs detected in shallow sequencing approach which attributed to *L. amylovorus* taking up a large fraction of the SAGs (412 SAGs) in the data. Therefore, more SAGs can compensate for fewer reads per SAG in shallow sequencing compared to with other less dominant bacterial species.

## Conclusion

Single-cell metagenomics sequencing, when combined with bulk sequencing approaches for the entire microbial community, enables the study of microbial community heterogeneity in the environment by revealing cell-to-cell variability. Our study demonstrated that different sequencing strategies in single-cell metagenomics can yield varying results, where one must choose between higher sampled diversity but lower genome coverage, or lower diversity with higher genome coverage depending on the research objective. Despite underrepresentation of bacterial phyla due to the prerequisite for DNA in the sample to be cell-contained and to the number of selected droplets for sequencing, this technology remains valuable for exploring potential novel bacterial species that cannot be discovered through shotgun metagenomic sequencing. For investigating the acquisition of antibiotic resistance and the transmission of ARGs within the gut microbiome or environmental contexts (such as sewage environments), single-cell sequencing has proven to be a useful tool. The single-cell metagenomics analysis directly links ARGs genes to their bacterial hosts, as demonstrated in our study, could provide new insights into the complexity of ARGs evolution, dispersal, and emergence in real-life environments. Instead of simply copying the sequencing depth and number of SPCs used here, researchers would be able to determine an appropriate number of cells at an appropriate depth to accommodate their goals and sequence. It takes significantly more reads to consistently hit a single ARG, which means estimating resistance prevalence in low-abundance species requires more sequencing efforts.

## Data Availability

The datasets presented in this study can be found in online repositories. The names of the repository/repositories and accession number(s) can be found in the article/Supplementary material.

## Footnotes

^1^ https://emea.support.illumina.com/downloads/bcl2fastq-conversion-software-v2-20.html
