# Supplementary material for: High throughput single cell metagenomic sequencing with semi-permeable capsules: unraveling microbial diversity at the single-cell level in sewage and fecal microbiomes
Source: Front Microbiol. 2025 Feb 4;15:1516656. doi: 10.3389/fmicb.2024.1516656 (PMC11834865; doi:10.3389/fmicb.2024.1516656)
Supplement: Supplementary file 1 [file Supplementary_file_1.docx]

Supplementary materials for

High throughput Single Cell Metagenomic Sequencing with Semi-Permeable Capsules: Unraveling Microbial Diversity at the Single-Cell Level in Sewage and Fecal Microbiomes

Meilee Ling*^,1^, Judit Szarvas*^,1^, Vaida Kurmauskaitė^2^, Vaidotas Kiseliovas^2^, Rapolas Žilionis^2,3^, Baptiste Avot^1,4^, Patrick Munk^1^, Frank M. Aarestrup^1^

*shared first authorship

1: Research Group for Genomic Epidemiology, National Food Institute, Technical University of Denmark, Kgs Lyngby, Denmark
2: Atrandi Biosciences, Vilnius, Lithuania
3. Institute of Biotechnology, Life Sciences Center, Vilnius University, Lithuania
4: Imperial College London, United Kingdom

Supplementary data 1: Number of SPCs for each species found in the deep and shallow sequencing samples.

Supplementary data 2: Taxonomy assignment to each SPCs recovered in the deep and shallow sequencing.

Supplementary data 3: Count of AMR genes identified for each species in both deep and shallow sequencing samples.

 
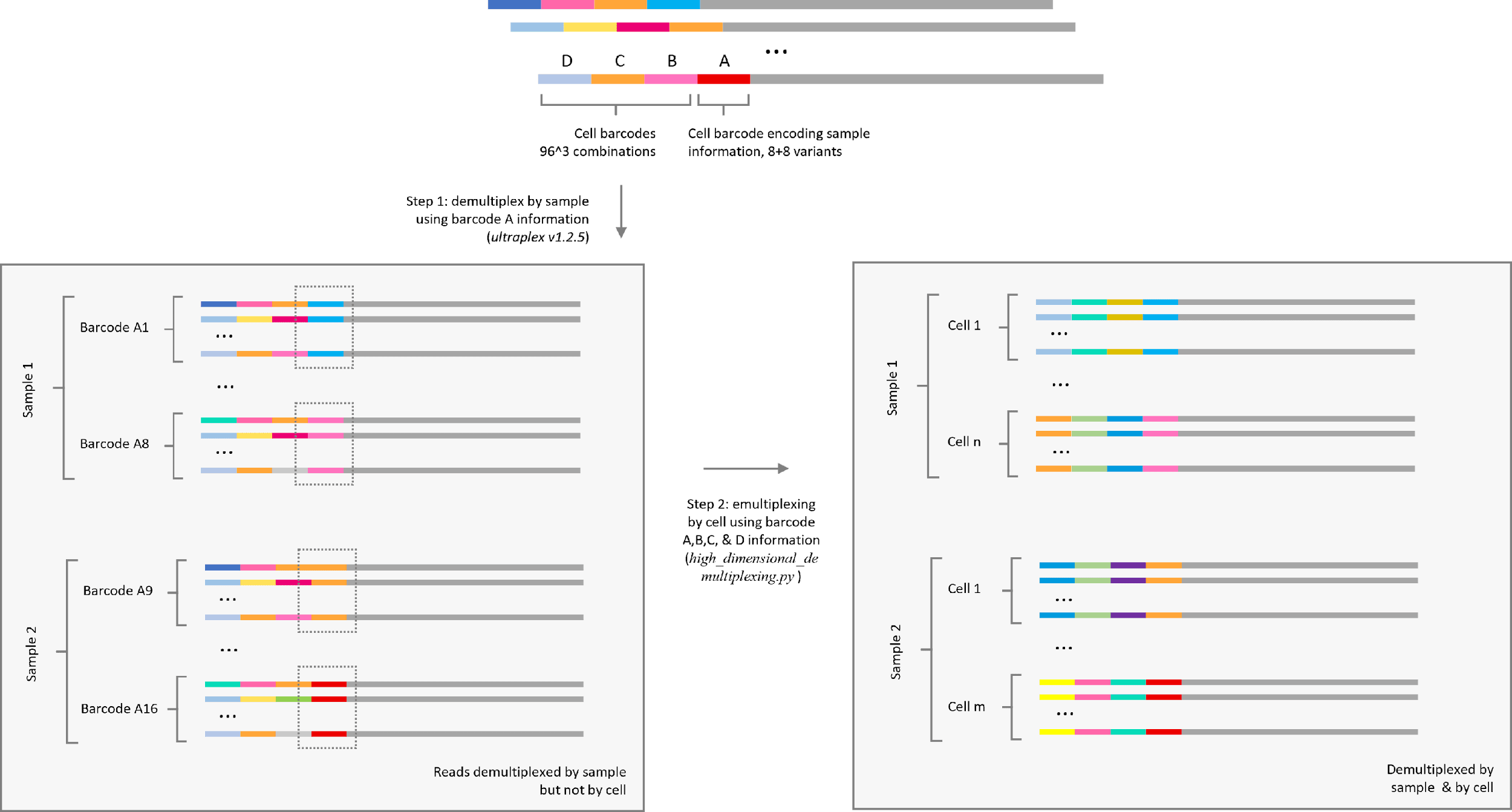


**Supplementary chart 1:** Read Demultiplexing Outline. The sequencing instrument outputs paired-end reads as two FASTQ files (R1.fastq and R2.fastq). The 5’ end of Read 2 contains the cell barcode information. The full cell barcode is composed of four barcodes, denoted as A, B, C, and D. In this study, barcode A was used to encode sample information (see Figure X); barcode A variants 1-8 were used for Sample 1, and barcode A variants 9-16 were used for Sample 2. There are 96 distinct variants of each barcode B, C, and D. The total barcode diversity is therefore 96 × 96 × 96 × 16. The first demultiplexing step involved grouping reads by barcode A information, which allowed them to also be split by sample. At the second demultiplexing step, each set of reads sharing the same barcode A variant underwent further demultiplexing by barcodes A+B+C to obtain per-cell demultiplexed data in the form of two FASTQ files (R1 and R2) for each cell. For simplicity, the schematic only shows the barcode-containing Read 2.

**Supplementary table 1:** Recovered Gram-negative and Gram-positive species in the spike in community in sewage sample (bgd01)

| **Species** | **Mapping depth** | **Template Coverage** | **SAG count** | **Theoretical genomic DNA (%)** | **Typing** |
| --- | --- | --- | --- | --- | --- |
| Clostridium perfringens | 10.46 | 65.80 | 1 | 0.0001 | Gram-Positive |
| Enterococcus faecalis | 5.56 | 0.93 | 0 | 0.0010 | Gram-Positive |
| Methanobrevibacter smithii | 0.09 | 1.94 | 0 | 0.1000 | *Archea* |
| Saccharomyces cerevisiae | 3.34 | 33.48 | 0 | 1.4000 | *Fungi* |
| Akkermansia muciniphila | 0.30 | 4.98 | 0 | 1.5000 | Gram-Negative |
| Candida albicans | 10.73 | 63.43 | 1 | 1.5000 | *Fungi* |
| Clostridioides difficile | 4.48 | 0.99 | 0 | 1.5000 | Gram-Positive |
| Bifidobacterium adolescentis | 696.20 | 99.14 | 63 | 6.0000 | Gram-Positive |
| Fusobacterium nucleatum | 0.22 | 4.20 | 0 | 6.0000 | Gram-Negative |
| Limosilactobacillus fermentum | 847.38 | 96.60 | 70 | 6.0000 | Gram-Positive |
| Prevotella corporis | 5.15 | 35.05 | 1 | 6.0000 | Gram-Negative |
| Bacteroides fragilis | 0.80 | 10.30 | 0 | 14.0000 | Gram-Negative |
| Escherichia coli | 4.45 | 33.43 | 0 | 14.0000 | Gram-Negative |
| Faecalibacterium prausnitzii | 24.56 | 90.30 | 4 | 14.0000 | Gram-Positive |
| Roseburia hominis | 426.33 | 99.69 | 25 | 14.0000 | Gram-Positive |
| Veillonella rogosae | 14.77 | 85.92 | 0 | 14.0000 | Gram-Negative |


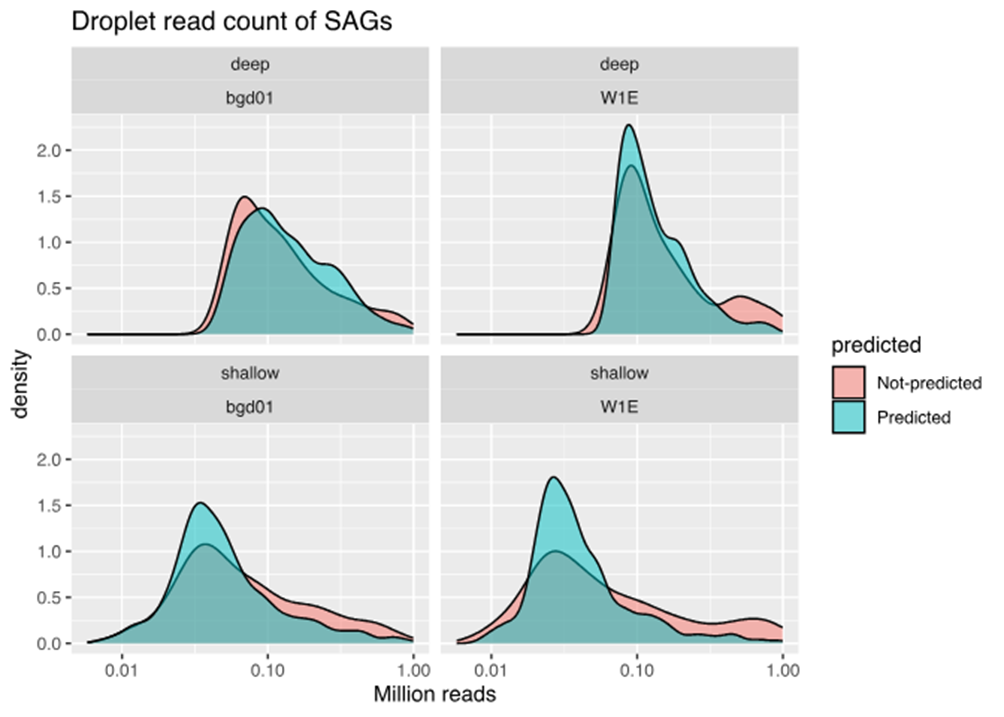


Supplementary figure 1: Distribution of read counts per SAG for the deep and shallow sequenced feces and sewage samples after 50,000 reads threshold filtered for bgd01 sample and 70,000 reads threshold filtered for W1E sample.


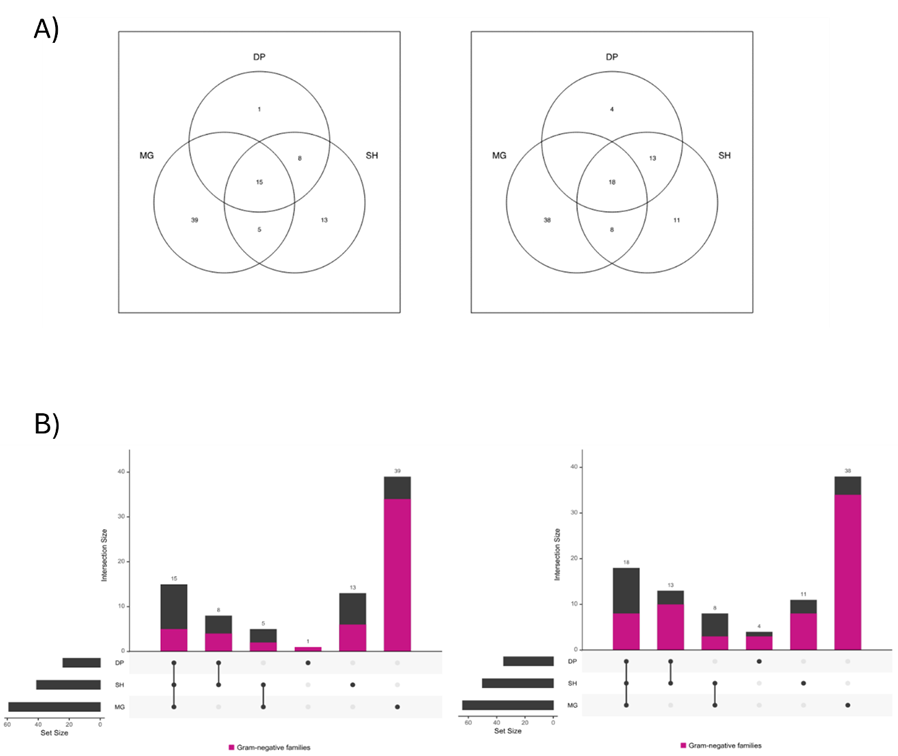


Supplementary figure 2: Panel A: Venn diagram showed the comparisons between the metagenome data and SC taxonomical results on numbers of bacteria families discovered. MG: Metagenomic data; DP: Deep sequencing; SH: Shallow sequencing. Left: W1E feces; Right: Sewage (bgd01). Panel B: Plots with Gram negative ratio with colored for the same data. Left: W1E feces; Right: Sewage (bgd01).
